# Balanced crystalloids *versus* saline for critically ill patients: an overview of systematic reviews

**DOI:** 10.62675/2965-2774.20260215

**Published:** 2026-05-20

**Authors:** Seok Woo Shin, Mayra Carvalho Ribeiro, Talita Magalhaes Sansoni, Francisco Vergueiro, Antonio Luis Eiras Falcao, Danilo da Silva Stamponi

**Affiliations:** 1 Department of Intensive Care Medicine Universidade Estadual de Campinas Campinas SP Brazil Department of Intensive Care Medicine, Universidade Estadual de Campinas - Campinas (SP), Brazil.; 2 Clinical Hospital Universidade Estadual de Campinas Campinas SP Brazil Clinical Hospital, Universidade Estadual de Campinas - Campinas (SP), Brazil.

**Keywords:** Balanced crystalloids, Normal saline, Critically ill patients

## Abstract

**Objective:**

To provide an overview of the evidence comparing the use of balanced crystalloids and normal saline in critically ill patients.

**Methods:**

A comprehensive literature search was conducted in PubMed®, Embase, and Cochrane databases through July 2024. Systematic reviews with meta-analyses comparing balanced crystalloids *versus* normal saline in critically ill patients were included. The methodological quality of the included reviews was assessed using the AMSTAR-2 tool.

**Results:**

Fourteen systematic reviews published between 2018 and 2024 met the inclusion criteria. Key clinical outcomes evaluated included mortality, acute kidney injury, and initiation of renal replacement therapy. Patient subgroups analyzed encompassed sepsis, trauma, hypovolemia, traumatic brain injury, postoperative status (cardiac and non-cardiac), and elderly populations. The methodological quality assessment of reviews using AMSTAR-2 revealed that most reviews had critical weaknesses in one or more domains. Quality appraisal revealed that one review had no critical domain weaknesses, while 13 reviews exhibited limitations in these domains. Evidence synthesis indicated a small benefit of balanced crystalloids compared to normal saline, especially among patients with sepsis and those without traumatic brain injury.

**Conclusion:**

This overview of systematic reviews suggests a small clinical advantage of balanced crystalloids over normal saline in critically ill patients, particularly in subgroups such as those with sepsis.

## INTRODUCTION

The intravenous administration of resuscitation fluids is a common intervention in the management of critically ill patients.^1^ Fluids are utilized to correct hypovolemia, support hemodynamic stabilization, and dilute medications. Their use may be beneficial in the treatment of circulatory shock, which represents the final common pathway of cardiovascular failure and is characterized by organ hypoperfusion. Fluid therapy can improve microvascular blood flow and increase cardiac output, thereby restoring tissue perfusion.^2^

Fluids are broadly categorized into crystalloids, including isotonic saline and balanced crystalloids, and colloids, with albumin being the most commonly used colloid solution.^3^ Physiological principles guide fluid selection and administration; however, clinical practice is largely influenced by availability and physician preference, resulting in significant regional variability.^4^

Given that isotonic saline may cause hyperchloremic metabolic acidosis - a condition associated with acute kidney injury (AKI)^1,5^- several randomized clinical trials (RCTs) and systematic reviews with meta-analyses have sought to compare the effects of balanced crystalloid solutions *versus* saline in critically ill patients. However, results regarding clinical outcomes have been inconsistent both across individual trials^6^ and in meta-analyses of systematic reviews.

The 2024 clinical practice guideline on fluid therapy in critically ill patients, published by the European Society of Intensive Care Medicine (ESICM), recommends balanced crystalloid solutions over isotonic saline for volume expansion.^7^ This guideline cites two systematic reviews with meta-analyses - Hammond et al.^8^ and Zampieri et al.,^9^ which both report limited evidence. Consequently, the recommendation is supported by low-quality evidence, and the panel’s suggestion was primarily based on the potential benefit of balanced crystalloids relative to isotonic saline.^7^

In specific subgroups - such as patients with sepsis, traumatic brain injury (TBI), and AKI - the evidence is less robust. The ESICM guideline recommends balanced crystalloids for critically ill patients with sepsis and AKI, with low and very low levels of evidence, respectively; conversely, it recommends isotonic saline for patients with TBI, based on very low-quality evidence. For patients with sepsis and septic shock, the 2021 international Surviving Sepsis Campaign guideline also recommends balanced crystalloids over saline; however, this recommendation is weak and supported by low-quality evidence.^10^

Despite numerous clinical trials and systematic reviews, the benefit of balanced crystalloid compared to saline remains uncertain. Several systematic reviews with meta-analyses have been published in recent years; however, no comprehensive synthesis summarizing the results of these meta-analyses currently exists. An overview of the available evidence could inform clinical decision-making by either supporting or challenging the 2024 clinical practice guideline recommendations.

No previous work has systematically aggregated evidence across multiple reviews while appraising their methodological quality. This study provides a comprehensive synthesis of outcomes across relevant subgroups, offering clarity and integration that prior guidelines did not address.

The present study aimed to provide an overview of the evidence comparing the use of balanced crystalloids and normal saline in critically ill patients.

## METHODS

This study provides an overview of systematic reviews with meta-analyses of RCTs and observational studies evaluating the use of balanced crystalloid solutions versus normal saline in critically ill patients. The methodology was based on the recommendations of the Cochrane Handbook for Systematic Reviews of Interventions for conducting overviews of reviews.^11^ The study protocol was prospectively registered in PROSPERO (International Prospective Register of Systematic Reviews) under registration number CRD42024580359.

### Literature search

A comprehensive literature search was conducted in PubMed®, Embase, and Cochrane databases up to July 2, 2024. The search strategy combined terms related to critically ill patients, balanced crystalloid solutions, normal saline, and fluid therapy. Detailed search strategies for each database are provided in table 1S (Supplementary Material).

### Eligibility criteria

Systematic reviews with meta-analyses of RCTs and observational studies comparing balanced crystalloid solutions *versus* normal saline in critically ill adult patients were included. Eligible reviews had to report on clinical outcomes such as mortality, AKI, or the need for renal replacement therapy (RRT). We excluded studies involving pediatric populations, narrative reviews, systematic reviews without meta-analyses, clinical trials, prospective observational studies, case reports or case series, conference abstracts, letters to the editor, clinical guidelines, theses, and dissertations. No restrictions were applied regarding publication date or language.

### Study selection

All records retrieved through the search were imported into Rayyan, a web-based tool for systematic reviews developed by the Qatar Computing Research Institute.^12^ After removing duplicates, two authors independently screened titles and abstracts to identify potentially eligible reviews. Full texts of selected articles were then independently assessed by the same authors against the eligibility criteria, with disagreements resolved by consensus.

### Quality assessment

The methodological quality of the included systematic reviews was assessed using the A Measurement Tool to Assess Systematic Reviews 2 (AMSTAR-2)^13^ by two authors independently, with any discrepancies resolved by consensus. Originally developed in 2007 for evaluating systematic reviews of randomized controlled trials, the AMSTAR tool was revised in 2017 to assess reviews that include non-randomized studies. AMSTAR-2 comprises 16 items, each rated as “Yes,” “Partial Yes,” or “No.” Critical domains, as defined by Shea et al., include items 2 (protocol registration before review commencement), 4 (adequacy of the literature search), 7 (justification for excluded studies), 9 (assessment of risk of bias in included studies), 11 (appropriateness of meta-analytical methods), 13 (consideration of risk of bias when interpreting results), and 15 (assessment of publication bias).^13^

### Certainty of evidence

Certainty of the review evidence was assessed using the Grading of Recommendations Assessment, Development and Evaluation (GRADE) approach.^14,15^Confidence in Network Meta-Analysis (CINeMA) web application was used to rate confidence in network meta-analysis.^16^ From each included systematic review, the GRADE or CINeMA ratings for the outcomes of mortality, AKI, and RRT were extracted when available.

### Assessment of overlap

To assess the overlap of primary studies across the included systematic reviews, a citation matrix was constructed, with rows representing the individual primary studies and columns representing the systematic reviews. Each cell indicated whether a primary study was included in a given review.

The degree of overlap was quantified using the Corrected Covered Area (CCA), calculated according to the method proposed by Pieper et al.^17^ The CCA is expressed as a percentage, with values interpreted as follows: slight (zero to 5%), moderate (6 - 10%), high (11 - 15%), and very high (> 15%). Higher values indicate greater redundancy among primary studies across the included reviews.

### Data extraction

From each included systematic review, the following data were extracted: author(s), year of publication, population and subgroups, intervention and control groups, study design, search strategy details (databases and last search date), number of included studies, primary and secondary outcomes, and results. Data extraction was performed using Google Sheets. Data extraction was independently performed by two authors, with any discrepancies resolved by consensus.

### Data synthesis

The characteristics and main findings of the systematic reviews, along with their methodological quality, were summarized in systematically structured tables. Meta-analytic measures of association with 95% confidence intervals (95%CIs) were reported as odds ratios (OR), relative risks (RR), mean differences (MD), or standardized mean differences (SMD). Heterogeneity among the included studies was also summarized; the I^2^ values correspond to those reported in each review, and no recalculation was performed.

The outcomes evaluated were mortality, AKI, the need for renal replacement RRT, major adverse kidney events at 30 days (MAKE30), changes in serum chloride concentration, length of hospital and intensive care unit (ICU) stay, ventilator-free days, and vasopressor-free days. The subgroups included patients with sepsis, trauma, hypovolemia, advanced age, TBI, and those undergoing cardiac or non-cardiac surgery.

### Ethics Committee approval

As this study is an overview of published systematic reviews and does not involve primary data collection with human participants, it did not require review or approval by an institutional ethics committee.

## RESULTS

### Search results

The literature search across PubMed®, Embase, and the Cochrane Library identified 3,454 potentially relevant records. After removing 79 duplicates, two authors independently screened titles and abstracts, leading to 14 articles selected for full-text assessment. Among these, one study was excluded because its design did not meet the criteria for a systematic review with meta-analysis, and another was excluded for an inappropriate study population. Ultimately, 12 studies met the eligibility criteria based on database searches.

**Figure 1:**
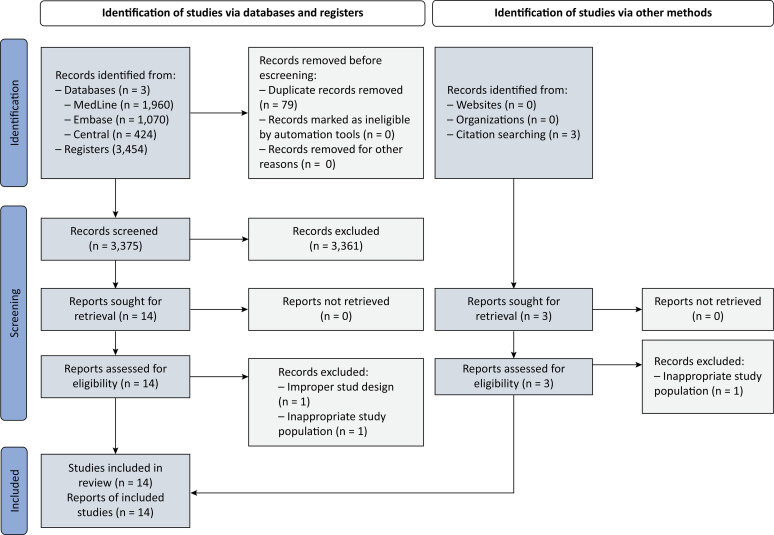
Study selection flowchart through literature search.

Citation tracking identified three additional records. Of these, one was excluded because of an inappropriate population, and two were included. Thus, a total of 14 systematic reviews were included in the final analysis. The study selection process is depicted in figure 1.

### Characteristics of systematic reviews

Table 1 summarizes the key characteristics of the 14 included systematic reviews. All were published in English between 2018 and 2024. Two reviews employed network meta-analysis methodologies (Liu et al.^18^; Tseng et al.^19^). Most reviews included only RCTs, except for Hammond et al.^20^ and González-Castro et al.,^21^ which also included observational studies. The number of individual studies included per review varied based on their design, search strategy, and eligibility criteria, with total patient populations ranging from 19,105 to 40,910.

**Table 1:** Characteristics of systematic reviews with meta-analyses

Author	Design	Studies included	Population	Intervention	Comparator	Outcome measure	Subgroups
Hammond et al.^(8)^	Meta-analysis and systematic review of RCTs	13 RCTs	35,884 critically ill adults	Balanced crystalloids	Normal saline	Mortality, AKI, RRT, MV-free days, vasopressor-free days	Sepsis, trauma, traumatic brain injury, and patients admitted to the ICU after cardiac surgery
Zampieri et al.^(9)^	Meta-analysis and systematic review of RCTs	6 RCTs	34,685 critically ill adults	Balanced crystalloids	Normal saline	In-hospital mortality, survival at last follow-up, RRT, days alive and out of hospital, and out of ICU	Surgical, sepsis, traumatic brain injury
Liu et al.^(18)^	Network meta-analysis and systematic review	49 RCTs	40,910 critically ill patients	Balanced crystalloids	Normal saline	Mortality, AKI, RRT	Sepsis, trauma, hypovolemia, elderly
Tseng et al.^(19)^	Network meta-analysis and systematic review	58 RCTs	26,351 critically ill patients	Balanced crystalloids	Normal saline	Mortality, fluid resuscitation volume, AKI, and transfusion volume	Sepsis, surgical, trauma, traumatic brain injury
Hammond et al.^(20)^	Meta-analysis of cohort studies and randomized trials	13 studies	30,950 critically ill patients	Balanced crystalloids	Normal saline	28-/30-days/ hospital mortality, ICU mortality, ICU length of stay, hospital length of stay, AKI, RRT, MV-free days, MAKE30, serum chloride concentration change	Sepsis
González-Castro et al.^(21)^	Meta-analysis of controlled, randomized clinical trials and prospective studies	8 studies	20,684 critically ill patients -adults (age 18 years or over) admitted to the ICU	Balanced crystalloids	Normal saline	Mortality in the ICU or hospital, AKI	No subgroups
Zayed et al.^(22)^	Meta-analysis and systematic review of RCTs	6 RCTs	19,332 critically ill patients	Balanced crystalloids	Normal saline	AKI, in-hospital mortality, ICU mortality, RRT	No subgroups
Chua et al.^(23)^	Meta-analysis and systematic review of RCTs	4 RCTs	19,105 critically ill patients	Balanced crystalloids	Normal saline	Mortality, AKI, RRT	No subgroups
Dong et al.^(24)^	Meta-analysis and systematic review of RCTs	8 RCTs	35,456 critically ill patients	Balanced crystalloids	Normal saline	Mortality, AKI, RRT	Sepsis, non-sepsis, TBI, non-TBI
Jackson et al.^(25)^	Meta-analysis and systematic review of RCTs	7 RCTs	20,171 critically ill patients	Balanced crystalloids	Normal saline	ICU and hospital mortality, MAKE30, AKI, RRT	No subgroups
Liu et al.^(26)^	Meta-analysis and systematic review of RCTs	9 RCTs	20,526 critically ill patients (> 18 years old) requiring fluid resuscitation	Balanced crystalloids	Normal saline	Mortality, AKI, RRT	No subgroups
Xue et al.^(27)^	Meta-analysis and systematic review of RCTs	8 RCTs	19,301 critically ill patients (acutely ill adult patients in the ICU or surgical adult patients transferred to the ICU in the perioperative period)	Balanced crystalloids	Normal saline	In-hospital mortality, AKI, RRT, RRT-free days, MAKE30, serum chloride concentration change, MV-free days, vasopressor-free days	Sepsis, non-sepsis, TBI, non-TBI
Zwager et al.^(28)^	Meta-analysis and systematic review of RCTs	9 RCTs	32,777 critically ill adults (intensive care medicine and emergency medicine)	Balanced crystalloids	Normal saline	Mortality, AKI, RRT	Sepsis, TBI
Chen et al.^(29)^	Meta-analysis and systematic review of RCTs	18 RCTs	36,224 critically ill adults	Balanced crystalloids	Normal saline	Composite mortality, AKI, RRT, hospital length of stay, MV-free days	Sepsis, trauma, TBI, admission to the ICU after surgery, diabetic ketoacidosis, and renal complications

RCT - randomized clinical trial; AKI - acute kidney injury; RRT - renal replacement therapy; MV - mechanical ventilation; ICU - intensive care unit; TBI - traumatic brain injury; MAKE30: composite outcome assessing major adverse kidney events within 30 days, including mortality, initiation of renal replacement therapy, and persistent renal dysfunction.

The most commonly reported clinical outcomes were mortality, AKI, and the need for RRT. Mortality was assessed in all 14 reviews, AKI in 13, and RRT in 12. Additional outcomes included MAKE30, changes in serum chloride concentration, length of hospital and ICU stay, ventilator-free days, and vasopressor-free days.

Subgroup analyses were conducted in clinically relevant populations, including patients with sepsis, trauma, hypovolemia, advanced age, TBI, and those undergoing cardiac or non-cardiac surgery.

In total, 30 primary studies (24 RCTs and 6 observational studies) assessing balanced crystalloid solutions and normal saline were included in the systematic reviews (Table 2S - Supplementary Material).

### Quality assessment

Methodological quality was assessed using the AMSTAR-2 tool, as detailed in table 2. Thirteen reviews presented critical methodological weaknesses, particularly in item 7, which concerns the description and justification of excluded studies. The quality appraisal indicated that only one review presented no critical weaknesses across the assessed domains, while thirteen displayed methodological limitations in these areas.

**Table 2:** The quality assessment results of systematic reviews included using the AMSTAR-2 tool

Author	AMSTAR-2 item																	Overall
	1	2*	3	4*	5	6	7*	8	9*	10	11*	12	13*	14	15*	16	Number of critical domains not met	
Chen et al.^(8)^	Y	N	Y	N	Y	Y	N	PY	Y	N	Y	Y	Y	Y	Y	Y	3	Critically low
Zampieri et al.^(9)^	Y	Y	Y	PY	Y	Y	N	Y	Y	N	Y	Y	Y	Y	Y	Y	1	Low
Liu et al.^(18)^	Y	Y	Y	Y	Y	Y	N	PY	Y	N	Y	Y	Y	Y	Y	Y	1	Low
Tseng et al.^(19)^	Y	Y	Y	PY	Y	Y	Y	Y	Y	N	Y	Y	Y	Y	Y	Y	0	High
Hammond et al.^(20)^	Y	Y	Y	Y	Y	Y	N	Y	Y	N	Y	Y	Y	Y	Y	Y	1	Low
González-Castro et al.^(21)^	Y	N	Y	Y	Y	Y	N	PY	Y	N	Y	Y	Y	Y	Y	Y	2	Critically low
Zayed et al.^(22)^	Y	N	Y	Y	N	Y	N	PY	Y	N	Y	Y	Y	Y	N	Y	3	Critically low
Chua et al.^(23)^	Y	N	Y	Y	N	N	N	PY	Y	N	Y	Y	Y	Y	N	Y	3	Critically low
Dong et al.^(24)^	Y	Y	Y	PY	Y	Y	N	Y	Y	N	Y	Y	Y	Y	Y	Y	1	Low
Jackson et al.^(25)^	Y	N	Y	Y	Y	Y	N	PY	Y	N	Y	Y	Y	Y	Y	Y	2	Critically low
Liu et al.^(26)^	Y	Y	Y	Y	Y	Y	N	PY	Y	N	Y	Y	Y	Y	Y	Y	1	Low
Xue et al.^(27)^	Y	Y	Y	PY	Y	Y	N	Y	Y	N	Y	Y	Y	Y	Y	Y	1	Low
Zwager et al.^(28)^	Y	Y	Y	Y	Y	Y	N	Y	Y	N	Y	Y	Y	Y	Y	Y	1	Low

Item 1: did the research questions and inclusion criteria for the review include the components of patient/population, intervention, comparison, and outcomes? Item 2: did the report of the review contain an explicit statement that the review methods were established before the conduct of the review, and did the report justify any significant deviations from the protocol? Item 3: did the review authors explain their selection of the study designs for inclusion in the review? Item 4: did the review authors use a comprehensive literature search strategy? Item 5: did the review authors perform study selection in duplicate? Item 6: did the review authors perform data extraction in duplicate? Item 7: did the review authors provide a list of excluded studies and justify the exclusions? Item 8: did the review authors describe the included studies in adequate detail? Item 9: did the review authors use a satisfactory technique for assessing the risk of bias (RoB) in individual studies that were included in the review? Item 10: did the review authors report on the sources of funding for the studies included in the review? Item 11: if a meta-analysis was performed, did the review authors use appropriate methods for the statistical combination of results? Item 12: if meta-analysis was performed, did the review authors assess the potential impact of RoB in individual studies on the results of the meta-analysis or other evidence synthesis? Item 13: did the review authors account for RoB in individual studies when interpreting/discussing the results of the review? Item 14: did the review authors provide a satisfactory explanation for, and discussion of, any heterogeneity observed in the results of the review? Item 15: if they performed quantitative synthesis, did the review authors carry out an adequate investigation of publication bias (small study bias) and discuss its likely impact on the results of the review? Item 16: did the review authors report any potential sources of conflict of interest, including any funding they received for conducting the review?

AMSTAR-2 indicates A Measurement Tool to Assess Systematic Reviews. N, no; PY, partial yes; RoB, risk of bias; Y, yes. *Critical domains.

### Certainty of evidence

The certainty of evidence for the outcomes of mortality, AKI, and RRT varied across the included systematic reviews. Eight studies assessed the certainty of evidence using the GRADE approach, whereas one network meta-analysis evaluated certainty using the CINeMA web application. Five reviews (Zayed et al.,^22^ Chua et al.,^23^ González-Castro et al.,^21^ Hammond et al.,^20^ and Dong et al.^24^) did not report any formal assessment of the certainty of evidence.

According to Liu et al.,^18^ Jackson et al.,^25^ and Liu et al.,^26^ the certainty of the evidence was rated as moderate for all three outcomes; however, the reviews did not specify the domains or reasons for downgrading. In contrast, Xue et al.^27^ reported low certainty for mortality and AKI, and very low certainty for RRT, due to risk of bias, imprecision, and publication bias. Zwager et al.^28^ rated the certainty as very low for mortality owing to inconsistency, indirectness, and publication bias, and low for AKI and RRT due to inconsistency and indirectness.

Among the more recent reviews, Hammond et al.^8^ found high certainty for mortality, moderate for AKI (downgraded due to inconsistency in AKI definitions), and low for RRT (downgraded for imprecision and heterogeneity). Chen et al.^29^ reported moderate certainty across all outcomes, citing risk of bias as the reason for downgrading. Similarly, Zampieri et al.^9^ rated the evidence for mortality and RRT as moderate, with downgrading for risk of bias in cluster-randomized trials and for inconsistency, respectively, while AKI was not assessed.

Using the CINeMA web application, Tseng et al.^19^ reported variable certainty across subgroups. For mortality, certainty was moderate for sepsis patients (downgraded for within-study bias), low for surgical patients (bias and imprecision), and very low for trauma and TBI patients (bias, imprecision, and heterogeneity). For AKI, certainty was low in sepsis and surgical patients and very low in trauma patients, with downgrading due to within-study bias, heterogeneity, imprecision, and incoherence. Renal replacement therapy was not assessed in that review.

The summary of the certainty of evidence is presented in table 3S (Supplementary Material).

### Assessment of overlap

The degree of overlap among the included reviews was quantified, and a citation matrix (Table 2S - Supplementary Material) was constructed to calculate the CCA, which was found to be 23.3%.

### Outcome assessment

#### Hospital mortality

All studies, except Tseng et al.,^19^ included data on hospital mortality in critically ill patients. Tseng et al. did not report overall mortality in critically ill patients, presenting mortality data only for subgroups (sepsis, trauma, TBI, and surgical patients). Hammond et al.^8^ separately analyzed a group of studies with low risk of bias for clinical outcome assessment.

Three systematic reviews reported statistically significant results favoring the use of balanced crystalloid solutions. These are: Liu et al.^18^ (OR 0.91; 95%CI 0.83 - 0.99; I^2^ = 8%), González-Castro et al.^21^ (adjusted OR 0.92; 95%CI 0.85 - 0.99; I^2^ = 0%; 8 individual studies with 20,684 patients), and Hammond et al.^20^ (RR 0.86; 95%CI 0.75 - 0.99; p = 0.04; I^2^ = 63%; 10 studies, including 4 observational, with 31,116 patients).

Detailed results are presented in table 3.

**Table 3:** Results of outcomes

Author	Mortality in-hospital	ICU mortality	Acute kidney injury	New renal replacement therapy	RRT-free days	MAKE30	Hospital length of stay	ICU length of stay	MV-free days	Vasopressor-free days	Serum chloride concentration changes
Hammond et al.^(8)^	Low risk of bias: RR 0.96; 95%CI 0.91 - 1.01; p = 0.13; I^2^ = 12.1%; 6 RCTs; 34,450 patients Overall: RR 0.93; 95%CI 0.76 - 1.15; p = 0.52; I^2^ = 88.4%; 11 RCTs	Not assessed	Low risk of bias: RR 0.96; 95%CI 0.89 - 1.02; p = 0.20; I^2^ = 8.6%; 5 RCTs; 25,224 patients Overall: RR 0.96; 95%CI 0.83 - 1.11; p = 0.54; I^2^ = 72.6%; 10 RCTs	Low risk of bias: RR 0.95; 95%CI 0.81 - 1.11; p = 0.53; I^2^= 59.5%; 5 RCTs; 33,554 patients Overall: RR 0.91; 95%CI 0.66 - 1.24; p = 0.54; I^2^ = 86.5%; 9 RCTs	Not assessed	Not assessed	Not assessed	Not assessed	MD 0.18; 95%CI -0.45 - 0.81; p = 0.58; I^2^ = 79.5%; 5 RCTs; 32,191 patients	MD 0.19; 95%CI -0.13 - 0.51]; p = 0.25; I^2^ = 24.1%; 3 RCTs; 21,62 2 patients	Not assessed
Zampieri et al.^(9)^	OR 0.96; 95%CI 0.91 - 1.02; 6 RCTs; 34,479 patients	Not assessed	Not assessed	OR 0.93; 95%CI 0.85 - 1.02; 6 RCTs; 33,431 patients	Not assessed	Not assessed	Not assessed	Not assessed	Not assessed	Not assessed	Not assessed
Liu et al.^(18)^	OR 0.91; 95%CI 0.83 - 0.99; I^2^ = 8%	Not assessed	OR 0.98; 95%CI 0.81 - 1.15; I^2^ = 7%	OR 0.96; 95%CI 0.74 - 1.45; I^2^ = 15%	Not assessed	Not assessed	Not assessed	Not assessed	Not assessed	Not assessed	Not assessed
Tseng et al.^(19)^	Not assessed	Not assessed	Not assessed	Not assessed	Not assessed	Not assessed	Not assessed	Not assessed	Not assessed	Not assessed	Not assessed
Hammond et al.^(20)^	RR 0.86; 95%CI 0.75 - 0.99; p = 0.04; I^2^ = 63%; 6 RCTs + 4 observational studies; 31,116 patients	RR 0.90; 95%CI 0.82 - 0.98; p = 0.02; I^2^ = 0%; 5 RCTs; 1,759 patients	RR 0.91; 95%CI 0.85 - 0.98; p = 0.007; I^2^ = 0%; 5 RCTs + 3 observational studies; 29,255 patients	RR 0.93; 95%CI 074-1.17; p = 0.55; I^2^ = 38%; 3 RCTs + 3 observational studies; 24,566 patients	Not assessed	Not assessed	MD 0.27 days; 95% CI [-0.47, 0.70]; p = 0.57; I^2^ = 52%; 3 RCTs + 2 observational studies	MD 0.00 days; 95% CI [-0.11, 0.12]; p = 0.98; I^2^ = 0%; 3 RCTs + 2 observational studies	MD 0.31 days; 95% CI [0.04, 0.57]; p = 0.02; I^2^ = 0%; 3 RCTs	Not assessed	MD -7.40; 95%CI -11.78 - -3.01; p = 0.0009; I^2^ = 82%; 1 RCT + 2 observational studies
González-Castro et al.^(21)^ (adjusted OR)	OR 0.92; 95%CI 0.85 - 0.99; I^2^ = 0%; 8 RCTs; 20,684 patients	Not assessed	OR 1.00; 95%CI 0.99 - 1.01; I^2^ = 0%; 7 RCTs	Not assessed	Not assessed	Not assessed	Not assessed	Not assessed	Not assessed	Not assessed	Not assessed
Zayed et al.^(22)^	OR 0.92; 95%CI 0.85 - 1.01; p = 0.09; I^2^ = 0%; 5 RCTs	OR 0.90; 95%CI 0.81 -1.01; p = 0.08; I^2^ = 0%; 4 RCTs	OR 0.92; 95%CI 0.84 - 1.01; p = 0.1; I^2^ = 0%; 5 RCTs	OR 0.92; 95%CI 0.67 - 1.28; p = 0.65; I^2^ = 38%; 5 RCTs	Not assessed	Not assessed	Not assessed	Not assessed	Not assessed	Not assessed	Not assessed
Chua et al.^(23)^	RR 0.91; 95%CI 0.82 -1.01; p = 0.09; I^2^ = 0%; 4 RCTs; 19,105 patients	Not assessed	RR 0.94; 95%CI 0.87 - 1.02; p = 0.17; I^2^ = 0%; 4 RCTs; 18,147 patients	RR 0.91; 95%CI 0.77 - 1.07; p = 0.26; I^2^ = 19%; 4 RCTs; 18,319 patients	Not assessed	Not assessed	Not assessed	Not assessed	Not assessed	Not assessed	Not assessed
Dong et al.^(24)^	RR 0.96; 95%CI 0.92 - 1.01; p = 0.09; I^2^ = 0%; 7 RCTs; 34,517 patients	Not assessed	RR 0.95; 95%CI 0.90 - 1.01; p = 0.08; I^2^ = 0%; 7 RCTs; 24,593 patients	RR 0.93; 95%CI 0.86 - 1.02; p = 0.11; I^2^ = 19%; 7 RCTs; 33,830 patients	Not assessed	Not assessed	Not assessed	Not assessed	Not assessed	Not assessed	Not assessed
Jackson et al.^(25)^	RR 0.92; 95%CI 0.85 - 1.00; p = 0.05; I^2^ = 0%; 6 RCTs; 20,105 patients	RR 0.91; 95%CI 0.82 - 1.00; p = 0.06; 6 RCTs; 20,125 patients	RR 0.94; 95%CI 0.86 - 1.02; p = 0.11; I^2^ = 0%; 3 RCTs; 18,082 patients	RR 0.91; 95%CI 0.77 - 1.027; p = 0.26; I^2^ = 19%; 4 RCTs; 18,319 patients	Not assessed	RR 0.95; 95%CI 0.88 - 1.01; p = 0.11; I^2^ = 0%; 4 RCTs; 18,933 patients	Not assessed	Not assessed	Not assessed	Not assessed	Not assessed
Liu et al.^(26)^	RR 0.93; 95%CI 0.86 - 1.01; p = 0.08; I^2^ = 0%; 8 RCTs; 20,345 patients	Not assessed	RR 0.94; 95%CI 0.88 - 1.00; p = 0.06; I^2^ = 0%; 7 RCTs; 19,203 patients	RR 0.94; 95%CI 0.69 - 1.27; p = 0.67; I^2^ = 39%; 5 RCTs	Not assessed	Not assessed	Not assessed	Not assessed	Not assessed	Not assessed	Not assessed
Xue et al.^(27)^	RR 0.92; 95%CI 0.85 - 1.00; p = 0.06; I^2^ = 0%; 8 RCTs; 19,301 patients	Not assessed	RR 0.93; 95%CI 0.86 - 1.01; p = 0.09; I^2^ = 0%; 6 RCTs; 18,261 patients	RR 0.91; 95%CI 0.77 - 1.07; p = 0.24; I^2^ = 20%; 5 RCTs; 18,379 patients	SMD 0.09; 95%CI 0.06 - 0.12; p < 0.00001; I^2^ = 0%; 2 RCTs	RR 0.93; 95%CI 0.88 - 1.00; p = 0.04; I^2^ = 0%; 2 RCTs; 16,696 patients	Not assessed	Not assessed	SMD 0.08, 95%CI 0.05 - 0.11; p < 0.001; I^2^ = 0%; 3 RCTs; 16,822 patients	SMD 0.04, 95%CI 0.00 - 0.07; p = 0.02; I^2^ = 0%; 2 RCTs; 16,776 patients	SMD -1,23; 95%CI -1.59 - -0.87; p < 0.00001; I^2^ = 0%; 3 RCTs
Zwager et al.^(28)^	RR 0.94; 95%CI 0.82 - 1.07; p = 0.36; I^2^ = 0%; 5 RCTs; 6,350 patients	Not assessed	RR 0.95; 95%CI 0.86 - 1.06; p = 0.40; I^2^ = 0%; 6 RCTs; 6,417 patients	RR 0.87; 95%CI 0.63 - 1.20; p = 0.41; I^2^ = 9%; 5 RCTs; 6,401 patients	Not assessed	Not assessed	Not assessed	Not assessed	Not assessed	Not assessed	Not assessed
Chen et al.^(29)^	RR 0.96; 95%CI 0.93 - 1.00; p = 0.03; I^2^ = 0%; 18 RCTs; 36,244 patients	RR 0.97; 95%CI 0.87 - 1.08; p = 0.57; 6 RCTs; 23,992 patients	RR 0.93; 95%CI 0.87 - 0.99; p = 0.03; I^2^ = 0%; 8 RCTs; 28,918 patients	RR 0.95; 95%CI 0.83 - 1.08; p = 0.34; I^2^ = 0%; 7 RCTs; 23,294 patients	Not assessed	Not assessed	SMD -0.07; 95% CI [-0.41, 0.27]; p = 0.64; I^2^ = 39%; 7 RCTs; 357 patients	Not assessed	SMD 0.03; 95%CI -0.01 - 0.07; p = 0.10; I^2^ = 0%; 3 RCTs; 21,363 patients	Not assessed	Not assessed

ICU - intensive care unit; RRT - renal replacement therapy; MV - mechanical ventilation; RR - relative risk; MD - mean difference; RCT - randomized clinical trial.

#### Intensive care unit mortality

Three systematic reviews reported on ICU mortality. The study by Zayed et al.^22^included four RCTs and found an OR of 0.90 (95%CI 0.81 - 1.01; p = 0.08; I^2^ = 0%). Hammond et al.^20^ analyzed five RCTs with 1,759 patients, reporting a RR of 0.90 (95%CI 0.82 - 0.98; p = 0.02; I^2^ = 0%). Chen et al.^29^ included six RCTs with 23,992 patients, finding an RR of 0.97 (95%CI 0.87 - 1.08; p = 0.57).

The results for ICU mortality are summarized in table 3.

#### Acute kidney injury

Twelve reviews assessed AKI (Table 3). Two reported significant benefits with balanced crystalloids. Hammond et al.^20^ reported a RR of 0.91 (95%CI 0.85 - 0.98; p = 0.007; I^2^ = 0%) across eight studies, including three observational studies, with a total of 29,255 patients. Similarly, Chen et al.^29^ found an RR of 0.93 (95%CI 0.87 - 0.99; p = 0.03; I^2^ = 0%) across eight studies with 28,918 patients.

#### Renal replacement therapy

Thirteen reviews evaluated the RRT requirement, with no significant difference found between balanced crystalloid and saline groups (Table 3).

#### Renal replacement therapy-free days

Only Xue et al.^27^ assessed RRT-free days, reporting a benefit with balanced crystalloids (SMD 0.09; 95%CI 0.06 - 0.12; p < 0.00001; I^2^ = 0%) (Table 3).

#### Major adverse kidney events within 30 days

Jackson et al.^25^ and Xue et al.^27^ included MAKE30 in their systematic reviews. No significant difference was observed between balanced crystalloid solutions and normal saline regarding this outcome (Table 3).

#### Hospital length of stay

Hospital length of stay was assessed in two systematic reviews, by Hammond et al.^20^ and Chen et al.^29^No significant difference was observed (Table 3).

#### Intensive care unit length of stay

Only Hammond et al.^20^ evaluated this outcome (5 studies: 3 RCTs, 2 observational). No difference was found (Table 3).

#### Mechanical ventilation-free days

The outcomes of MV-free days were reported in four reviews: Xue et al.,^27^ Hammond et al.,^20^ Hammond et al.,^8^ and Chen et al.^29^ Two showed significant benefit (Table 3).

The study by Xue et al.^27^ included three RCTs with 16,822 patients, with an SMD of 0.08 days (95%CI 0.05 - 0.11; p < 0.001; I^2^ = 0%). The study by Hammond et al.^20^ included three RCTs, with an MD of 0.31 days (95%CI 0.04 - 0.57; p = 0.02; I^2^ = 0%).

#### Vasopressor-free days

Xue et al.^27^ and Hammond et al.^8^ reported no difference between groups (Table 3).

#### Serum chloride concentration

Two reviews found a significantly smaller increase in serum chloride with balanced crystalloids. These studies were Xue et al.^27^ (SMD -1.23; 95%CI -1.59 to -0.87); p < 0.00001; I^2^ = 0%) and Hammond et al.^20^ MD -7.40; 95%CI -11.78 to -3.01); p = 0.0009; I^2^ = 82%) (Table 3).

## Outcomes assessment in different subgroups

### Sepsis and non-sepsis patients

Mortality in the sepsis subgroup was assessed in nine systematic reviews, of which four reported a significant benefit with balanced crystalloids.

The studies by Xue et al.^27^ (RR 0.86; 95%CI 0.75 - 0.98; p = 0.02; I^2^ = 0%), Zwager et al.^28^ (RR 0.87; 95%CI 0.77 - 0.98; p = 0.02; I^2^ = 0%), Tseng et al.^19^ (OR 0.84; 95%CI 0.74 - 0.95; p < 0.01), and Chen et al.^29^ (RR 0.91; 95%CI 0.85 - 0.99; p = 0.02) showed statistically significant results.

Acute kidney injury in this subgroup was assessed in one review by Tseng et al.^19^ with no difference noted.

The need for RRT was evaluated in the review by Zampieri et al.,^9^ which found no benefit of using a balanced crystalloid solution.

The composite outcome of MAKE30 was reported in two reviews, by Xue et al.^27^and Hammond et al.,^20^ with RR 0.81 (95%CI 0.66 - 1.01; p = 0.21; I^2^ = 61%) and OR 0.78 (95%CI 0.66 - 0.91; p = 0.002; I^2^ = 42%), respectively.

Mortality in non-sepsis patients was assessed in 4 studies (Xue et al.,^27^ Zwager et al.,^28^Dong et al.,^24^ and Zampieri et al.^9^), with no significant difference (Table 4).

**Table 4:** Outcome results for mortality, acute kidney injury, renal replacement therapy, and MAKE30 in sepsis patients, and mortality in non-sepsis patients, traumatic brain injury patients, and non-traumatic brain injury patients

Author	Mortality (sepsis)	AKI (sepsis)	RRT (sepsis)	MAKE30 (sepsis)	Mortality (non-sepsis)	Mortality (TBI)	Mortality (non-TBI)
Hammond et al.^(8)^	Low risk of bias: RR 0.93; 95%CI 0.86 - 1.01; p = 0.10; I^2^ = 22.3%; 5 RCTs; 6,754 patients Overall: RR 0.93; 95%CI 0.85 - 1.01; p = 0.07; I^2^ = 19.3%; 6 RCTs	Not assessed	Not assessed	Not assessed	Not assessed	RR 1.26; 95%CI 0.98 - 1.60; p = 0.07; I^2^ = 20.2%; 3 RCTs; 1,896 patients	Not assessed
Zampieri et al.^(9)^	OR 0.95; 95%CI 0.88 - 1.03; 6 RCTs; 6,753 patients	Not assessed	OR 0.88; 95%CI 0.77 - 1.00; 6 RCTs; 6,686 patients	Not assessed	OR 0.98; 95%CI 0.92 - 1.05; 6 RCTs; 27,707 patients	OR 1.36; 95%CI 1.09 - 1.70; 6 RCTs; 1,961 patients	OR 0.95; 95%CI 0.90 - 1.00; 6 RCTs; 32,502 patients
Liu et al.^(18)^	OR 1.01; 95%CI 0.46 - 2.10; I^2^ = 20%	Not assessed	Not assessed	Not assessed	Not assessed	Not assessed	Not assessed
Tseng et al.^(19)^	OR 0.84; 95%CI 0.74 - 0.95; p < 0.01; moderate confidence rating	OR 0.98; 95%CI 0.82 - 1.17; low confidence rating	Not assessed	Not assessed	Not assessed	OR 1.29; 95%CI 0.33 - 5.03; very low confidence rating	Not assessed
Hammond et al.^(20)^	OR 0.98; 95%CI 0.80 - 1.20; p = 0.86; I^2^ = 0%; 1 RCT + 2 observational studies; 7,292 patients	Not assessed	Not assessed	OR 0.78; 95%CI 0.66 - 0.91; p = 0.002; I^2^ = 42%; 2 RCTs; 939 patients	Not assessed	Not assessed	Not assessed
González-Castro et al.^(21)^	Not assessed	Not assessed	Not assessed	Not assessed	Not assessed	Not assessed	Not assessed
Zayed et al.^(22)^	Not assessed	Not assessed	Not assessed	Not assessed	Not assessed	Not assessed	Not assessed
Chua et al.^(23)^	Not assessed	Not assessed	Not assessed	Not assessed	Not assessed	Not assessed	Not assessed
Dong et al.^(24)^	RR 0.94; 95%CI 0.87 - 1.00; p = 0.04; I^2^ = 0%; 4 RCTs; 6,501 patients	Not assessed	Not assessed	Not assessed	RR 0.98; 95%CI 0.92 - 1.04; p = 0.46; I^2^ = 0%; 4 RCTs; 26,925 patients	RR 1.24; 95%CI 1.02 - 1.50; p = 0.03; I^2^ = 7%; 3 RCTs; 1,906 patients	RR 0.94; 95%CI 0.90 - 0.99; p = 0.02; I^2^ = 0%; 4 RCTs; 31,534 patients
Jackson et al.^(25)^	Not assessed	Not assessed	Not assessed	Not assessed	Not assessed	Not assessed	Not assessed
Liu et al.^(26)^	Not assessed	Not assessed	Not assessed	Not assessed	Not assessed	Not assessed	Not assessed
Xue et al.^(27)^	RR 0.86; 95%CI 0.75 - 0.98; p = 0.02; I^2^ = 0%; 2 RCTs; 2,420 patients	Not assessed	Not assessed	RR 0.81; 95%CI 0.66 - 1.01; p = 0.21; I^2^ = 61%; 2 RCTs; 2,596 patients	RR 0.96; 95%CI 0.86 - 1.07; p = 0.48; I^2^ = 0%; 5 RCTs; 15,794 patients	RR 1.11; 95%CI 0.86 - 1.43; p = 0.43; I^2^ = 0%; 2 RCTs; 1,420 patients	RR 0.90; 95%CI 0.82 - 0.99; p = 0.02; I^2^ = 0%; 5 RCTs; 16,794 patients
Zwager et al.^(28)^	RR 0.87; 95%CI 0.77 - 0.98; p = 0.02; I^2^ = 0%; 2 RCTs; 1,873 patients	Not assessed	Not assessed	Not assessed	RR 0.96; 95%CI 0.82 - 1.11; p = 0.59; I^2^ = 0%;2 RCTs; 5,692 patients	Not assessed	Not assessed
Chen et al.^(29)^	RR 0.91; 95%CI 0.85 - 0.99; p = 0.02; 6 RCTs; 6,914 patients	Not assessed	Not assessed	Not assessed	Not assessed	RR 1.26; 95%CI 0.93 - 1.70; p = 0.13; 4 RCTs; 1,927 patients	Not assessed

AKI - acute kidney injury; RRT - renal replacement therapy; TBI - traumatic brain injury; RR – relative risk; RCT - randomized clinical trial; OR – odds ratio.

### Traumatic brain injury and non-traumatic brain injury patients

Mortality in TBI patients was reported in six reviews, with two reaching statistical significance: Dong et al.^24^ (RR 1.24; 95%CI 1.02 - 1.50; p = 0.03; I^2^ = 7%) and Zampieri et al.^9^ (OR 1.36; 95%CI 1.09 - 1.70). These studies suggest a potential benefit of isotonic saline (Table 4).

Mortality in non-TBI patients was described in three reviews, with two reaching statistical significance: Xue et al.^27^ (RR 0.90; 95%CI 0.82 - 0.99; p = 0.02; I^2^ = 0%) and Dong et al.^24^ (RR 0.94; 95%CI 0.90 - 0.99; p = 0.02; I^2^ = 0%).

### Post-trauma and postoperative patients

No significant differences in mortality or AKI were observed in patients admitted after trauma or surgery. Similar findings were reported for cardiac surgery (Hammond et al.^8^) (Table 5).

**Table 5:** Outcome results for mortality and acute kidney injury in intensive care unit patients admitted after trauma and surgery (cardiac or non-cardiac), and mortality in patients with hypovolemia, advanced age, acute kidney injury, and diabetic ketoacidosis

Author	Mortality (trauma)	AKI (trauma)	Mortality (surgical)	AKI (surgical)	Mortality (after cardiac surgery)	Mortality (hypovolemia)	Mortality (elderly)	Mortality (AKI)	Mortality (DKA)
Hammond et al.^(8)^	Low risk of bias: RR 0.93; 95%CI 0.86 - 1.01; p = 0.10; I^2^ = 22.3%; 5 RCTs; 6,754 patients Overall: RR 0.93; 95%CI 0.85 - 1.01; p = 0.07; I^2^ = 19.3%; 6 RCTs	Not assessed	Not assessed	Not assessed	Not assessed	Not assessed	Not assessed	Not assessed	Not assessed
Zampieri et al.^(9)^	OR 0.95; 95%CI 0.88 - 1.03; 6 RCTs; 6,753 patients	Not assessed	OR 0.88; 95%CI 0.77-1.00; 6 RCTs; 6,686 patients	Not assessed	OR 0.98; 95%CI 0.92 - 1.05; 6 RCTs; 27,707 patients	Not assessed	Not assessed	Not assessed	Not assessed
Liu et al.^(18)^	OR 0.45; 95%CI 0.07 - 2.15; I^2^ = 20%	Not assessed	Not assessed	Not assessed	Not assessed	OR 0.90; 95%CI 0.32 - 2.52; I^2^ = 22%	OR 1.00; 95%CI 0.03 - 28.55; I^2^ = 89%	Not assessed	Not assessed
Tseng et al.^(19)^	OR 0.84; 95%CI 0.74 - 0.95; p < 0.01; moderate confidence rating	OR 0.98; 95% CI 0.82 - 1.17; low confidence rating	Not assessed	Not assessed	Not assessed	Not assessed	Not assessed	Not assessed	Not assessed
Hammond et al.^(20)^	OR 0.98; 95%CI 0.80 - 1.20; p = 0.86; I^2^ = 0%; 1 RCT + 2 observational studies; 7,292 patients	Not assessed	Not assessed	OR 0.78; 95%CI 0.66-0.91; p = 0.002; I^2^ = 42%; 2 RCTs; 939 patients	Not assessed	Not assessed	Not assessed	Not assessed	Not assessed
González-Castro et al.^(21)^	Not assessed	Not assessed	Not assessed	Not assessed	Not assessed	Not assessed	Not assessed	Not assessed	Not assessed
Zayed et al.^(22)^	Not assessed	Not assessed	Not assessed	Not assessed	Not assessed	Not assessed	Not assessed	Not assessed	Not assessed
Chua et al.^(23)^	Not assessed	Not assessed	Not assessed	Not assessed	Not assessed	Not assessed	Not assessed	Not assessed	Not assessed
Dong et al.^(24)^	RR 0.94; 95%CI 0.87 - 1.00; p = 0.04; I^2^ = 0%; 4 RCTs; 6,501 patients	Not assessed	Not assessed	Not assessed	RR 0.98; 95%CI 0.92-1.04; p = 0.46; I^2^ = 0%; 4 RCTs; 26,925 patients	Not assessed	Not assessed	Not assessed	Not assessed
Jackson et al.^(25)^	Not assessed	Not assessed	Not assessed	Not assessed	Not assessed	Not assessed	Not assessed	Not assessed	Not assessed
Liu et al.^(26)^	Not assessed	Not assessed	Not assessed	Not assessed	Not assessed	Not assessed	Not assessed	Not assessed	Not assessed
Xue et al.^(27)^	RR 0.86; 95%CI 0.75 - 0.98; p = 0.02; I^2^ = 0%; 2 RCTs; 2,420 patients	Not assessed	Not assessed	RR 0.81; 95%CI 0.66 - 1.01; p = 0.21; I^2^ = 61%; 2 RCTs; 2,596 patients	RR 0.96; 95%CI 0.86 - 1.07; p = 0.48; I^2^ = 0%; 5 RCTs; 15,794 patients	Not assessed	Not assessed	Not assessed	Not assessed
Zwager et al.^(28)^	RR 0.87; 95%CI 0.77 - 0.98; p = 0.02; I^2^ = 0%; 2 RCTs; 1,873 patients	Not assessed	Not assessed	Not assessed	RR 0.96; 95%CI 0.82 - 1.11; p = 0.59; I^2^ = 0%; 2 RCTs; 5,692 patients	Not assessed	Not assessed	Not assessed	Not assessed
Chen et al.^(29)^	RR 0.91; 95%CI 0.85 - 0.99; p = 0.02; 6 RCTs; 6,914 patients	Not assessed	Not assessed	Not assessed	Not assessed	Not assessed	Not assessed	RR 0.98; 95%CI 0.92 - 1.04; p = 0.46; 4 RCTs; 5,607 patients	RR 0.78; 95%CI 0.24 - 2.49; p = 0.67; 3 RCTs; 149 patients

AKI - acute kidney injury; DKA - diabetic ketoacidosis.

### Hypovolemia, advanced age, acute kidney injury, and diabetic ketoacidosis

Hypovolemic and elderly patients were evaluated in Liu et al.,^18^ while patients with AKI and diabetic ketoacidosis were analyzed in the study by Chen et al.^29^ No mortality differences were found in these subgroups (Table 5).

## Summary of evidence across systematic reviews

Table 6 provides a consolidated summary of the key clinical outcomes assessed across all included systematic reviews. For each major endpoint, the table aggregates the number of systematic reviews that evaluated it, the approximate total number of patients, the direction and consistency of effects, and the certainty of the evidence when reported.

**Table 6:** Summary of evidence across systematic reviews

Outcome	No. of systematic reviews	Approx. total patients	Direction of effect	Consistency across reviews	Certainty of evidence (if reported)
Mortality	13	~6,000 - 35,000	↓ ↔ Small reduction or neutral	High consistency	Low–moderate
Mortality in sepsis	9	~2,000 - 7,000	↓ ↔ Small reduction or neutral	Moderate consistency	Low
Mortality in TBI	6	~1,400 - 2,000	↑↔ Small increase or neutral	Moderate consistency	Very low
Mortality in non-TBI	3	~16,000 - 32,000	↓ ↔ Small reduction or neutral	High consistency	Very low
Acute kidney injury	12	~6,000 - 30,000	↔	High consistency	Low–moderate
Renal replacement therapy	13	~6,000 - 34,000	↔	Low–moderate consistency	Very low–low

↓ = reduced (favors balanced crystalloids); ↔ = no significant difference; ↑ = increased (favors saline); TBI - traumatic brain injury.

## DISCUSSION

This overview synthesized data from 14 systematic reviews with meta-analyses comparing balanced crystalloid solutions and normal saline in critically ill patients. The cumulative evidence indicates a small clinical advantage of balanced crystalloids, particularly among subgroups such as patients with sepsis and those without TBI. Although most reviews reported non-significant results, those that did show significant effects consistently favored balanced crystalloids. Across outcomes, balanced crystalloids were associated with trends toward reduced mortality, lower AKI incidence, more RRT-free and mechanical ventilation-free days, and smaller increases in serum chloride concentration.

Several reviews, including Chua et al.,^23^ Hammond et al.,^8^ Chen et al.,^29^ and Zampieri et al.,^9^ reported non-significant but clinically relevant trends suggesting potential benefits of balanced crystalloids. Bayesian analyses further supported a probability of mortality reduction, even when traditional frequentist statistics did not reach significance. Overall, this synthesis suggests that balanced crystalloids may offer incremental benefits in critically ill populations, though the magnitude of effect remains modest and uncertain.

Subgroup analyses revealed heterogeneity in treatment effects. In sepsis, balanced crystalloids were generally associated with improved outcomes, while in TBI populations, isotonic saline appeared to be more favorable - possibly reflecting pathophysiological differences in intracranial dynamics.^30^ In other subgroups, such as trauma, postoperative, or hypovolemic patients, no consistent differences were observed.

### Methodological strengths and limitations

This overview has several methodological strengths, including the comprehensive search across major databases, inclusion of only systematic reviews with meta-analyses, and quantitative assessment of review overlap. However, limitations intrinsic to the umbrella review methodology should be acknowledged.

First, umbrella reviews rely exclusively on secondary data, which limits control over the quality and consistency of included primary studies. Consequently, any methodological weaknesses, publication bias, or selective reporting present in the original trials may be propagated or even amplified in this synthesis.

Second, overlap of primary studies across reviews can result in duplication of evidence and potential bias in pooled estimates. Although overlap was formally quantified using the CCA (23.3%), this value exceeds the commonly accepted threshold of 15%, indicating moderate-to-high overlap and reduced independence of evidence sources. This limitation underscores the need for cautious interpretation of aggregated results.

Third, heterogeneity in the methodological rigor, search strategies, and reporting standards of included reviews further complicates comparison and synthesis. The AMSTAR-2 appraisal revealed that most reviews had at least one critical weakness, particularly regarding the justification of excluded studies. These factors collectively reduce confidence in the strength and consistency of conclusions.

The methodological choices made in this overview - such as the inclusion of both pairwise and network meta-analyses, and the decision to quantify review overlap - enhance transparency and rigor. Nevertheless, if overlap analysis had not been performed, it would have limited the ability to evaluate evidence independence, a common challenge in umbrella reviews. Even with overlap quantification, the high CCA highlights the complexity of synthesizing evidence in fields with numerous overlapping meta-analyses. Future overviews might benefit from sensitivity analyses that weight reviews according to quality or degree of overlap to mitigate this issue.

The certainty of evidence across reviews was predominantly moderate to low for mortality and AKI, and low to very low for RRT. Variability in how certainty assessments were conducted - particularly incomplete application or reporting of GRADE criteria - limits cross-review comparability. Only one review used the CINeMA framework, underscoring the need for greater methodological standardization in future meta-research. To discuss the evaluation of evidence certainty in network meta-analyses, we referred to the conceptual framework proposed by Salanti et al.,^16^ which underpins the CINeMA approach. CINeMA evaluates the confidence in results from network meta-analyses across six domains - within-study bias, reporting bias, indirectness, imprecision, heterogeneity, and incoherence - building upon the GRADE principles to provide a structured and transparent assessment of evidence quality. Transparent, domain-specific downgrading and consistent use of these frameworks would enhance interpretability and confidence in pooled estimates.

### Implications and recommendations for future research

Given the observed limitations, future research should prioritize:

1. Standardized definitions and reporting of outcomes, particularly for AKI, are needed to improve cross-study comparability.
2. Individual patient data meta-analyses to reduce bias from study-level aggregation and enable more nuanced subgroup analyses.
3. Comprehensive application of GRADE or CINeMA in all evidence syntheses to enhance transparency in certainty assessment.
4. Explicit evaluation and management of overlap in future umbrella reviews, possibly through quality-weighted synthesis or exclusion of redundant reviews.

In summary, while balanced crystalloids appear to offer potential clinical advantages over isotonic saline in critically ill patients, the overall certainty of evidence remains limited by methodological heterogeneity, overlapping data, and reliance on secondary analyses. Future high-quality, standardized research is warranted to confirm these findings and clarify the contexts in which balanced solutions provide the greatest benefit.

## CONCLUSION

The findings of this overview of systematic reviews suggest a small benefit of using balanced crystalloid solutions over normal saline in critically ill patients, particularly in those with sepsis and those without traumatic brain injury.

## SUPPLEMENTARY MATERIAL

SUPPLEMENTARY MATERIAL
