# Supplementary material for: Balanced crystalloids versus saline for critically ill patients: an overview of systematic reviews
Source: Crit Care Sci. 2026 May 20;38:e20260215. doi: 10.62675/2965-2774.20260215 (PMC13399232; doi:10.62675/2965-2774.20260215)
Supplement: SUPPLEMENTARY MATERIAL [file 2965-2774-ccsci-38-e20260215-suppl01.pdf]

Critical Care Science
https://doi.org/10.62675/2965-2774.20260215

SUPPLEMENTARY MATERIAL

Balanced crystalloids *versus* saline for critically ill patients: an overview of systematic reviews

Seok Woo Shin[1], Mayra Carvalho Ribeiro[2], Talita Magalhaes Sansoni[1], Francisco Vergueiro Neto[1], Antonio Luis Eiras Falcao[1], Danilo da Silva Stamponi[1]

Table 1S - Search strategies

Database	Search strategy	Records
Pubmed	#1 (((((“Critical illness”[MeSH Terms]) OR (“Critical illness”[Title/Abstract])) OR (“Critical Illnesses”[Title/Abstract])) OR (“Illness, Critical”[Title/Abstract])) OR (“Illnesses, Critical”[Title/Abstract])) OR (“Critically Ill”[Title/Abstract]) 83,950<br>#2 (((((“Crystalloid solutions”[MeSH Terms]) OR (“Crystalloid solutions”[Title/Abstract])) OR (“Solutions, Crystalloid”[Title/Abstract])) OR (“Crystalloid Solution”[Title/Abstract])) OR (“Solution, Crystalloid”[Title/Abstract])) OR (“Crystalloid”[Title/Abstract]) 9,774<br>#3 ((((((“Saline solution”[MeSH Terms]) OR (“Saline solution”[Title/Abstract])) OR (“Normal Saline”[Title/Abstract])) OR (“Saline, Normal”[Title/Abstract])) OR (“0.9% Saline”[Title/Abstract])) OR (“Saline, 0.9%”[Title/Abstract])) OR (“0.9% NaCl”[Title/Abstract]) 52,412<br>#4 ((((((“Fluid therapy”[MeSH Terms]) OR (“Fluid therapy”[Title/Abstract])) OR (“Therapy, Fluid”[Title/Abstract])) OR (“Fluid Therapies”[Title/Abstract])) OR (“Therapies, Fluid”[Title/Abstract])) OR (“Rehydration”[Title/Abstract])) OR (“Rehydrations”[Title/Abstract]) 31,962<br>#5 (((“balanced crystalloid”[Title/Abstract]) OR (“balanced solution”[Title/Abstract])) OR (“balanced multielectrolyte solution”[Title/Abstract])) OR (“plasma-lyte”[Title/Abstract]) 531<br>#6 #2 OR #3 OR #4 OR #5 88,939<br>#7 #1 AND #6 1,960	1,960
Embase	(‘critical illness’/exp OR ‘critical illness’) AND (‘crystalloid’/exp OR ‘crystalloid’ OR ‘crystalloid formation’ OR ‘crystalloid solution’ OR ‘crystalloid solutions’ OR ‘sodium chloride’/exp OR ‘alcathion’ OR ‘bacteriostatic sodium chloride 0.9%’ OR ‘broncho saline’ OR ‘dendritis’ OR ‘flexivial’ OR ‘gingivyl’ OR ‘halite’ OR ‘hypersal’ OR ‘hypertonic lactated saline solution’ OR ‘hypertonic saline’ OR ‘hypertonic saline bath’ OR ‘hypertonic sodium chloride’ OR ‘hypertonic sodium chloride solution’ OR ‘hyposaline’ OR ‘hypotonic sodium chloride’ OR ‘hypotonic sodium chloride solution’ OR ‘natrium chloride’ OR ‘natural saline’ OR ‘normal saline’ OR ‘physiological saline’ OR ‘physiological solution’ OR ‘purex’ OR ‘saline’ OR ‘saline solution’ OR ‘saline solution, hypertonic’ OR ‘salt’ OR ‘sodium chloride’ OR ‘sodium chloride 0.45%’ OR ‘sodium chloride 0.9%’ OR ‘sodium chloride 23.4%’ OR ‘sodium chloride 3%’ OR ‘sodium chloride 5%’ OR ‘sodium chloride solution’ OR ‘sodiumchloride’ OR ‘table salt’ OR ‘fluid therapy’/exp OR ‘fluid therapy’ OR ‘parenteral fluid therapy’ OR ‘therapy, fluid’ OR ‘balanced crystalloid’ OR ‘balanced solution’ OR ‘acetic acid plus gluconate sodium plus magnesium chloride plus potassium chloride plus sodium chloride’/exp OR ‘acetic acid plus gluconate sodium plus magnesium chloride plus potassium chloride plus sodium chloride’ OR ‘magnesium chloride plus potassium chloride plus sodium acetate plus sodium chloride plus sodium gluconate’ OR ‘normosol’ OR ‘normosol r’ OR ‘normosol r ph 7.4’ OR ‘normosol-r’ OR ‘normosol-r ph 7.4’ OR ‘physiolyte’ OR ‘physiolyte physiosol physiosol ph 7.4 plasma-lyte a synovalyte’ OR ‘physiosol’ OR ‘physiosol ph 7.4’ OR ‘plasma-lyte’ OR ‘plasma-lyte 148’ OR ‘plasma-lyte 148 in water’ OR ‘plasma-lyte a’ OR ‘plasma-lyte a ph-7.4’ OR ‘plasmalyte’ OR ‘plasmalyte a’ OR ‘synovalyte’) 4,067<br>#1 AND [embase]/lim NOT ([embase]/lim AND [medline]/lim) 1,070	1,070

Continue...

This is an open access article under the CC BY license (https://creativecommons.org/licenses/by/4.0/).

...continuation

Cochrane	#1 MeSH descriptor: [Critical Illness] explode all trees 3,697<br>#2 Illnesses, Critical 635<br>#3 Critical Illnesses 635<br>#4 Critically Ill 9,789<br>#5 Illness, Critical 8,145<br>#6 {OR #1-#5} 14,367<br>#7 MeSH descriptor: [Crystalloid Solutions] explode all trees 1094<br>#8 Solutions, Crystalloid 899<br>#9 Crystalloid Solution 900<br>#10 Solution, Crystalloid 900<br>#11 Crystalloid 2,228<br>#12 MeSH descriptor: [Saline Solution] explode all trees 454<br>#13 Saline, Normal 21,555<br>#14 Saline, 0.9%6,427<br>#15 Normal Saline 21,555<br>#16 0.9% NaCl 2,008<br>#17 0.9% Saline 6,427<br>#18 MeSH descriptor: [Fluid Therapy] explode all trees 2244<br>#19 Therapy, Fluid 19,361<br>#20 Therapies, Fluid 1,443<br>#21 Fluid Therapies 1,443<br>#22 Rehydrations; Rehydration 1<br>#23 balanced crystalloid 326<br>#24 balanced solution 1,772<br>#25 balanced multielectrolyte solution 5<br>#26 plasma-lyte 176<br>#27 {OR #7-#26} 47408<br>#28 #6 AND #27 1,450	424 Cochrane Reviews
TOTAL		3,454

Table 2S - Citation matrix indicating overlap of primary studies among the included systematic reviews

		Systematic reviews														
		**Liu et al.**[18]	**González-Castro et al.**[21]	**Zayed et al.**[22]	**Chua et al.**[23]	**Jackson et al.**[25]	**Liu et al.**[26]	**Xue et al.**[27]	**Zwager et al.**[28]	**Hammond et al.**[20]	**Tseng et al.**[19]	**Dong et al.**[24]	**Hammond et al.**[8]	**Chen et al.**[29]	**Zampieri et al.**[9]	**Total references (duplicates)**
Randomized clinical trials	Waters 2001	0	0	0	0	1	0	1	0	1	1	0	1	1	0	6
	Takil 2002	0	0	0	0	0	0	1	0	0	0	0	0	0	0	1
	Mahler 2011	0	0	0	0	0	0	0	0	1	0	0	0	0	0	1
	Wu 2011	1	1	0	0	0	1	0	0	0	0	0	0	1	0	4
	Van Zyl 2012	1	0	0	0	0	0	1	1	0	0	0	0	1	0	4
	Annane 2013	0	1	0	0	0	1	0	0	1	1	0	0	1	0	5
	Rouquilly 2013	0	0	0	0	0	0	0	0	0	0	0	0	1	0	1
	Young 2014	1	1	1	0	1	1	1	1	1	1	1	1	1	0	12
	Young 2015	1	1	1	1	1	1	1	1	1	1	1	1	1	1	14
	Verma 2016	1	1	1	1	1	1	1	1	1	0	1	1	1	0	12
	Semler 2016	1	1	1	1	1	1	1	1	1	0	1	1	1	1	13
	Ratanarat 2017	0	0	1	0	0	1	0	1	0	0	1	1	0	0	5
	Reddy 2017	0	0	0	0	1	0	0	0	0	0	0	0	0	0	1
	Rossman 2017	0	0	0	0	0	0	0	0	0	0	0	0	1	0	1
	Choosakul 2018	0	0	0	0	0	1	0	1	0	0	0	1	1	0	4
	De-Madaria 2018	0	0	0	0	0	0	0	0	0	0	0	0	1	0	1
	Kunupakan 2018	0	0	0	0	0	0	0	0	0	0	0	1	0	0	1
	Self 2018	0	0	0	0	0	0	0	1	0	0	0	0	0	0	1
	Semler 2018	1	1	1	1	1	1	1	1	1	1	1	1	1	1	14
	Golla 2020	0	0	0	0	0	0	0	0	0	0	0	1	1	0	2
	Ramanan 2021	0	0	0	0	0	0	0	0	0	0	0	1	1	1	3
	Zampieri 2021	0	0	0	0	0	0	0	0	0	0	1	1	1	1	4
	Finfer 2022	0	0	0	0	0	0	0	0	0	0	1	1	1	1	4
	Karki 2022	0	0	0	0	0	0	0	0	0	0	0	0	1	0	1
Observational studies	Chua 2012	0	0	0	0	0	0	0	0	1	0	0	0	0	0	1
	Yunos 2012	1	0	0	0	0	0	0	0	0	0	0	0	0	0	1
	Haghunathan 2014	0	0	0	0	0	0	0	0	1	0	0	0	0	0	1
	Shaw 2015	0	0	0	0	0	0	0	0	1	0	0	0	0	0	1
	Kuca 2017	0	0	0	0	0	0	0	0	1	0	0	0	0	0	1
	Jaynes 2018	0	0	0	0	0	0	0	0	1	0	0	0	0	0	1
	N (total references, including duplicates)	8	7	6	4	7	9	8	9	13	5	8	13	18	6	121
	r (rows: number of unique primary studies)															30
	c (columns: number of included reviews)															14
	Corrected Covered Area															0,2333333333

Rows = primary studies; columns = systematic reviews; cells = study included (yes: 1, no: 0). The degree of overlap was quantified using the Corrected Covered Area (CCA), calculated as: , where N is the total number of included citations across all reviews, r is the number of unique primary studies, and c is the number of systematic reviews. Values closer to 0 indicate low overlap, while higher values indicate greater overlap. According to commonly used thresholds, overlap is interpreted as: slight (0 - 5%), moderate (6 - 10%), high (11 - 15%), and very high (> 15%).

Table 3S - Summary table of certainty of evidence (GRADE system or CINeMA web application)

Author	Mortality: quality of the evidence	Acute kidney injury: quality of the evidence	Renal Replacement Therapy use: quality of the evidence
Liu et al.[18]	⊕ ⊕ ⊕ ○ (Moderate - The review did not provide reasons for downgrading)	⊕ ⊕ ⊕ ○ (Moderate - The review did not provide reasons for downgrading)	⊕ ⊕ ⊕ ○ (Moderate - The review did not provide reasons for downgrading)
González-Castro et al.[21]	Not reported	Not reported	Not reported
Zayed et al.[22]	Not reported	Not reported	Not reported
Chua et al.[23]	Not reported	Not reported	Not reported
Jackson et al.[25]	⊕ ⊕ ⊕ ○ (Moderate - The review did not provide reasons for downgrading)	⊕ ⊕ ⊕ ○ (Moderate - The review did not provide reasons for downgrading)	⊕ ⊕ ⊕ ○ (Moderate - The review did not provide reasons for downgrading)
Liu et al.[26]	⊕ ⊕ ⊕ ○ (Moderate - The review did not provide reasons for downgrading)	⊕ ⊕ ⊕ ○ (Moderate - The review did not provide reasons for downgrading)	⊕ ⊕ ⊕ ○ (Moderate - The review did not provide reasons for downgrading)
Xue et al.[27]	⊕ ⊕ ○ ○ (Low due to the risk of bias, and imprecision)	⊕ ⊕ ○ ○ (Low due to the risk of bias, and imprecision)	⊕ ○ ○ ○ (Very low due to the risk of bias, imprecision, and publication bias)
Zwager et al.[28]	⊕ ○ ○ ○ (Very low due to inconsistency, indirectness, and publication bias)	⊕ ⊕ ○ ○ (Low due to inconsistency and indirectness)	⊕ ⊕ ○ ○ (Low due to inconsistency and indirectness)
Hammond et al.[20]	Not reported	Not reported	Not reported
Tseng et al.[19] (using CINeMA web application)	Sepsis patients - Moderate (within-study bias)<br>Surgical patients - Low (within-study bias and imprecision)<br>Trauma patients - Very low (within-study bias and imprecision)<br>Trauma brain injury patients - Very low (within-study bias, imprecision, and heterogeneity)	Sepsis patients - Low (within-study bias and heterogeneity)<br>Surgical patients - Low (imprecision)<br>Trauma patients - Very low (imprecision and incoherence)	Not reported
Dong et al.[24]	Not reported	Not reported	Not reported
Hammond et al.[8]	⊕ ⊕ ⊕ ⊕ (High)	⊕ ⊕ ⊕ ○ (Moderate - downgraded one level because of inconsistency in the definition of acute kidney injury)	⊕ ⊕ ○ ○ (Low - Downgraded two levels because of imprecision, evidenced by the wide confidence limits, and for inconsistency as evidenced by the heterogeneity (I^2 = 84.2%)
Chen et al.[29]	⊕ ⊕ ⊕ ○ (Moderate due to the risk of bias)	⊕ ⊕ ⊕ ○ (Moderate due to the risk of bias)	⊕ ⊕ ⊕ ○ (Moderate due to the risk of bias)
Zampieri et al.[9]	⊕ ⊕ ⊕ ○ (Moderate - downgraded 1 due to risk of bias in cluster randomised trials)	Not reported	⊕ ⊕ ⊕ ○ (Moderate - Downgraded 1 for inconsistency due to heterogeneity)
